# Modulation of Spheroid Forming Capacity and TRAIL Sensitivity by KLF4 and Nanog in Gastric Cancer Cells

**DOI:** 10.3390/cimb45010018

**Published:** 2022-12-30

**Authors:** Han Thi Ngoc To, Qui Anh Le, Hang Thi Thuy Bui, Ji-Hong Park, Dongchul Kang

**Affiliations:** 1 Ilsong Institute of Life Science, Hallym University, Beodeunaru-ro 55, Yeongdeungpo-gu, Seoul 07247, Republic of Korea; 2 Department of Biomedical Gerontology, Hallym University Graduate School, Chuncheon 24252, Republic of Korea

**Keywords:** gastric cancer, KLF4, Nanog, SOX2, OCT4, spheroid, TRAIL, cisplatin

## Abstract

The expression of pluripotency factors, and their associations with clinicopathological parameters and drug response have been described in various cancers, including gastric cancer. This study investigated the association of pluripotency factor expression with the clinicopathological characteristics of gastric cancer patients, as well as changes in the expression of these factors upon the stem cell-enriching spheroid culture of gastric cancer cells, regulation of sphere-forming capacity, and response to cisplatin and TRAIL treatments by Nanog and KLF4. Nanog expression was significantly associated with the emergence of a new tumor and a worse prognosis in gastric cancer patients. The expression of the pluripotency factors varied among six gastric cancer cells. KLF4 and Nanog were expressed high in SNU-601, whereas SOX2 was expressed high in SNU-484. The expression of KLF4 and SOX2 was increased upon the spheroid culture of SNU-601 (KLF4/Nanog-high) and SNU-638 (KLF4/Nanog-low). The spheroid culture of them enhanced TRAIL-induced viability reduction, which was accompanied by the upregulation of death receptors, DR4 and DR5. Knockdown and overexpression of Nanog in SNU-601 and SNU-638, respectively, did not affect spheroid-forming capacity, however, its expression was inversely correlated with DR4/DR5 expression and TRAIL sensitivity. In contrast, KLF4 overexpression in SNU-638 increased spheroid formation, susceptibility to cisplatin and TRAIL treatments, and DR4/DR5 expression, while the opposite was found in KLF4-silenced SNU-601. KLF4 is supposed to play a critical role in DR4/DR5 expression and responses to TRAIL and cisplatin, whereas Nanog is only implicated in the former events only. Direct regulation of death receptor expression and TRAIL response by KLF4 and Nanog have not been well documented previously, and the regulatory mechanism behind the process remains to be elucidated.

## 1. Introduction

More than a million cases of gastric cancer (5.6%, the fifth most frequent) were newly diagnosed and about 769,000 deaths (7.7%, the fourth leading cause of cancer death) were reported in 2020 worldwide [1]. East Asian nations, including China, Japan, and Korea, have seen a high prevalence of gastric cancer. Although the overall incidence of gastric cancer has decreased, it is rising in the population that is younger than 50 in both high and low-risk countries [2]. Recent advances in diagnosis, surgery, and therapy enable diagnosis at the early stage and have prolonged the overall survival of gastric cancer patients [2]. However, the prognosis of advanced and relapsed gastric cancer patients remains poor, necessitating sustained endeavors for improved diagnostic and therapeutic measures [3].

Cancer tissues consist of heterogeneous cells including cancer stem cells (CSCs) that are responsible for tumor initiation, metastasis, and resistance to conventional chemotherapy and radiotherapy [4]. CSCs were identified in both hematological cancers and various solid cancers including gastric, lung, breast, liver, and colorectal cancers [5]. CSCs were distinguished and verified by side population analysis, expression of various intracellular or cell surface CSC-specific markers, and tumor-forming capacity at the xenotransplantation of tumor cells [6]. Sphere-forming culture of cancer cells on ultra-low attachment plates was also employed to enrich the CSCs or CSC-like cells of certain solid cancers [7]. Resistance to conventional therapies and the tumor-initiating capacity of CSCs poses a serious threat in cancer treatment. Naturally, the identification and eradication of CSCs have been challenged through exhaustive investigations on the properties of the CSCs, including drug response and CSC-specific signaling pathways [8]. 

CSCs manifest similar characteristics shown in normal stem cells that continuously replenish tissue cells through incessant self-renewal and differentiation into diverse cell types [9]. Differentiated cells such as fibroblasts were transformed into induced pluripotent stem cells (iPSCs) that are functionally equivalent to embryonic stem cells (ESCs), by ectopic expression of pluripotency factors including OCT4, Nanog, SOX2, Myc, and KLF4 [10]. The expression of the pluripotency factors is critical to maintaining the self-renewal, undifferentiated state, and differentiation potential of ESCs. Pluripotency factors are also known to play important roles in the specification and maintenance of CSCs [11]. Incomplete reprogramming by transient expression of pluripotency factors in a mouse resulted in tumor formation in various tissues [12]. In addition, expression of OCT4, Nanog, SOX2, and Myc was reported in CSCs of various cancers, and their expressions were associated with the prognosis of the patients, although an oncogenic role of KLF4 appeared to be dependent on tissues [11,13]. 

The existence of CSCs in gastric cancer was reported in various studies [14]. The expression of pluripotency factors including Nanog, OCT4, and SOX2, and their association with prognosis, are also known in gastric cancer [15]. Expression of OCT4, SOX2, and Nanog, either alone or in combination was found positively associated with phenotypes of advanced gastric cancer and worse prognosis, while that of KLF4 was inversely correlated [16]. In addition, the spheroid culture of gastric cancer altered the expression of pluripotency factors, which might be associated with drug resistance and tumor formation in immunodeficient mice [17,18]. On the contrary, no association of Nanog, OCT4, and SOX2 with clinicopathological parameters was also reported in an analysis of tissue microarray of gastric cancer, which argues against their roles in gastric cancer progression [19]. Rather, a decrease or loss of KLF4 expression was observed in the late stage of cancer and proposed as a predictor for poor survival of patients. Therefore, an association of individual pluripotency factor expression with clinical specifications of gastric cancer patients is not firmly settled and remains to be clarified further. 

Resistance to conventional chemotherapy is a characteristic of CSCs to which expression of certain pluripotency factors can contribute either directly or indirectly [13,20,21,22]. Cisplatin (cis-diamminedichloroplatinum (II): CDDP) is one of the most widely used chemotherapeutic agents in cancer treatment, including gastric cancer [23,24]. Further, tumor necrosis factor-related apoptosis-inducing ligand (TRAIL) induces apoptosis of various tumor cells while sparing normal cells. The tumor-specific apoptosis-inducing activity of TRAIL has been exploited in its development into a tumor therapeutic, including gastric cancer [25]. The expression of OCT4 and Nanog was proposed to be positively associated with cisplatin resistance in ID1-silenced gastric cancer cells [26]. Direct silencing of OCT4, SOX2, and Nanog also increased cisplatin sensitivity in lung cancer cells, anaplastic thyroid cancer cells, and head and neck squamous cell carcinoma cells, respectively [27,28,29]. As in their oncogenic role, KLF4 showed a cell type-dependent effect on cisplatin sensitivity either by sensitizing colon cancer cell HCT-15 [30] or by heightening resistance in HepG2 hepatocarcinoma cells [31]. In contrast, the effect of direct modulation of the pluripotency factors on TRAIL sensitivity has not been clarified yet, although CSCs and cancer spheroid are known to be resistant to TRAIL-induced apoptosis [32].

This study aimed to define the role of pluripotency factors (OCT4, SOX2, Nanog, and KLF4) in gastric cancer progression, spheroid forming capacity, and drug resistance. An association of expression of the pluripotency factors at the transcript level with clinicopathological parameters of gastric cancer patients was analyzed here. In addition, changes in the expression of the pluripotency factors upon spheroid culture and treatment with cisplatin and TRAIL were examined in gastric cancer cells. The effect of KLF4 and Nanog expression on spheroid formation, and responses to cisplatin and TRAIL treatments, was investigated by either silencing or overexpressing their expression in two gastric cancer cells, SNU-601 (KLF4/Nanog-high) and SNU-638 (KLF4/Nanog-low).

## 2. Materials and Methods

### 2.1. Cell Lines and Cell Culture

Human gastric cancer cell lines, SNU-216, SNU-484, SNU-601, SNU-638, SNU-668, and SNU-719 were purchased from Korea Cell Line Bank (Seoul, Korea). All cell lines were cultured in RPMI-1640 (Gibco, Grand Islands, NY, USA) supplemented with 10% fetal bovine serum (Welgene, Daegu, Korea), 5% L-glutamine (Gibco), and 1% penicillin/streptomycin (Gibco), and maintained at 37 °C in 5% CO_2_-humidified atmosphere. The gastric cancer cell lines were maintained by passaging twice a week.

### 2.2. Tumor Spheroid Formation

Indicated cells (SNU-601, SNU-638, and their derivatives) were cultured in nonadherent conditions on six-well plates of ultralow attachment (Corning, Corning, NY, USA). Cells were seeded at a density of 3000 cells per well in serum-free RPMI-1640 medium plus 1% N-2 supplement (Gibco), 2% B-27 supplement (Gibco), 1% penicillin/streptomycin (Gibco), 20 ng/mL human FGF-2 (ProSpec, East Brunswick, NJ, USA), and 100 ng/mL EGF (ProSpec) (spheroid culture medium). The spheroid formation was observed 7 days and 14 days later, and pictures were taken with phase contrast microscopy (Olympus, Tokyo, Japan) at 40× magnification. Area taken by spheroids was analyzed with Image J software (ij153-win-java8, https://imagej.nih.gov, accessed on 23 June 2021).

### 2.3. MTT Assay

Cell viability was measured by the 3-(4,5-dimethylthiazol-2-yl)-2,5-diphenyltetrazoliumbromide (MTT) assay. Cells were seeded at a density of 4 × 10^3^ cells per well into 96-well plates with 100 µL culture medium (RPMI-1640) and treated as specified in the figure legends on the next day. At the indicated time, 20 µL of MTT solution (5 mg/mL in PBS, Sigma-Aldrich, St. Louis, MO, USA) was added and incubated further for 3 h. The resulting formazan crystal was dissolved by the addition of 80 µL MTT solubilizer (10% SDS in 0.01 N HCl). The absorbance was measured at 570 nm with reference absorbance at 650 nm with a Multiskan GO spectrophotometer (Thermo Scientific, Rockland, IL, USA).

### 2.4. Western Blot Analysis

Cells were lysed with radioimmunoprecipitation assay (RIPA) buffer of 50 mM Tris-HCl (pH 7.4), 0.1% SDS, 1% Triton X-100, 0.1% Nonidet P-40, and 0.5% sodium deoxycholate freshly supplemented with DTT (1 mM) and protease inhibitors (final concentration leupeptin 5 µg/mL; pepstatin A 2.5 µg/mL; aprotinin 5 µg/mL and PMSF 100 µM) and phosphatase inhibitors (100 µM Na3VO4, 100 µM NaF). Protein concentration was determined with BCA Protein Assay Reagent (Pierce, Rockford, IL, USA). Twenty-five µg protein per well was resolved in a 10% SDS-polyacrylamide gel and transferred to a PVDF membrane (Roche, Penzberg, Germany). The membrane was blocked with 5% nonfat dry milk in TBST (10 mM Tris-HCl [pH 7.6], 150 mM NaCl, and 0.1% Tween 20) for 1 h and incubated with a primary antibody overnight at 4 °C. The membrane was then incubated with either horseradish peroxidase (HRP)-conjugated goat anti-rabbit antibody or goat anti-mouse antibody (Pierce Biotech.) in TBST for 1 h at room temperature. After washing the membrane three times for five minutes each, the protein bands were detected by an ECL kit (Advansta, Menlo Park, CA, USA) and visualized with a ChemiDocTM MP System (BioRad, Hercules, CA, USA). Primary antibodies used were αNanog, αKLF4, αSOX2, αOCT4, αDR5 (Cell Signaling Tech., Danvers, MA, USA), αDR4 (Abnova Corp., Taipei, Taiwan), and αACTB (Santa Cruz Biotech, Dallas, TX, USA).

### 2.5. Flow Cytometry

Cells were collected by trypsinization and washed twice with phosphate-buffered saline (PBS). To measure the cell surface expression of CD44 and CD133, the collected cells were incubated with biotin-conjugated αCD44 antibody (eBioscience), FITC-conjugated streptavidin (eBioscience), and allophycocyanin (APC)-conjugated αCD133 antibody (Miltenyi Biotec, Bergisch Gladbach, Germany) for 30 min at RT in the dark. A mixture of mouse APC-conjugated IgG_1_ kappa isotype control, biotin-conjugated mouse IgG_1_ kappa isotype control, and FITC-conjugated streptavidin (all from eBioscience), was used as isotype control. FITC and APC fluorescence signals were determined by flow cytometry using FACSCalibur^TM^ (BD Bioscience, Sparks, MD, USA) and analyzed with Cell Quest Pro^TM^ software (version 5.2.1, BD Bioscience).

### 2.6. Silencing KLF4 and Nanog by Lentiviral shRNA Expression

The pLKO.1-based lentivirus vectors for short hairpin RNAs (shKLF4: TRCN0000010934 and shNANOG: TRCN0000004887) were purchased from Sigma-Aldrich, and pLKO-based scramble shRNA vector was obtained from Addgene (Cambridge, MA, USA). Lentiviruses were produced as previously described [33]. HEK293T cells were transfected with each lentiviral vector by the calcium phosphate precipitation method. The viral supernatant was collected after 48 h of transfection, filtered by 0.45 µm strain, and stored at −80 °C. The lentivirus was transduced into SNU-601 by adding corresponding virus particles mixed with polybrene (8 µg/mL, Sigma-Aldrich) to an incomplete medium free from serum, penicillin/streptomycin, and L-glutamine. After incubation for 8 h, the medium was changed to a fresh complete medium with 2 µg/mL puromycin for 7 days and selected cells were used for future experiments.

### 2.7. Overexpression of KLF4 and Nanog by Retroviral Transduction

Nanog and KLF4 ORF cDNAs were incorporated into pBABE-puro vectors. The ORF cDNA of Nanog was obtained by RT-PCR with forward (GGAATTCGCCACCATGAGTGTGGATCCAGCTTGT) and reverse (GCGTCGACTCACACGTCTTCAGGTTCGAT) primers with RNA extracted from SNU-601. The ORF cDNA of KLF4 was amplified from OSK vector (Addgene) with a primer set (forward: GGAATTCGCCACCATGGCTGTCAGCGACGCGCT, reverse: GCGTCGACTTAAAAATGCCTCTTCAT-GTGTAAGG). The pBABE-puro vector was used as a void control vector. Retroviruses were produced and used in experiments as lentivirus preparation which was described above.

### 2.8. Statistical Analysis

RNASeq data of gastric cancer tissue were downloaded from the TCGA database (stomach adenocarcinoma, PanCancer Atlas) through cBioPortal (http://www.cbioportal.org/, accessed on 23 June 2021), and analyzed by Wilcoxon rank sum test, Kaplan-Meier survival analysis and log-rank test using R project (R x64 4.1.2, https://www.r-project.org, downloaded on 3 November 2021). Experimental data were analyzed by two-tailed Student’s t-test and one-way ANOVA implemented in Microsoft Excel. Data were considered statistically significant when a *p*-value was smaller than 0.05.

## 3. Results

### 3.1. Pluripotency Factors in Gastric Cancer

**Figure 1:**
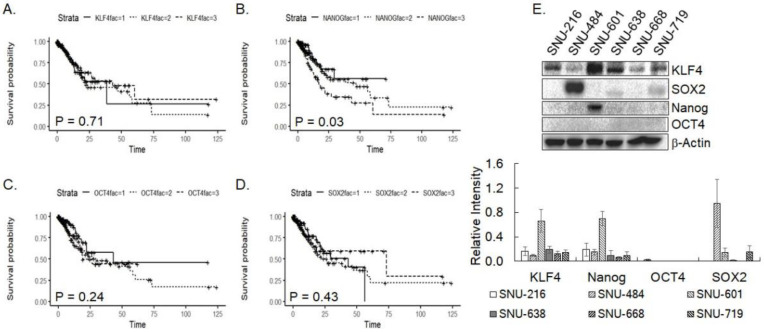
Pluripotency factors in gastric cancer. (**A**–**D**) Kaplan-Meier cumulative survival rate curve for four pluripotency factors, KLF4 (**A**), Nanog (**B**), OCT4 (**C**), and SOX2 (**D**). Solid lines represent expression lower than the first quartile (fac = 1), dotted lines for expression between the first and third quartile (fac = 2), and dashed lines for expression higher than the third quartile (fac = 3), respectively. (**E**) Expression of indicated pluripotency factors was examined in six gastric cancer cells by western blotting. Protein loading was monitored with β-Actin level against which quantified results were normalized. Data shown are mean ± SD of three independent experiments.

Expression of four pluripotency factors (OCT4, Nanog, SOX2, and KLF4) at the transcript level in gastric cancer tissue was analyzed against various pathological parameters using RNASeq data downloaded from the TCGA database (Table 1). While KLF4 expression was not associated significantly with any listed clinical parameters, significant associations of Nanog with new tumor emergence after initial treatment (*p* = 0.027), SOX2 with age (*p* = 0.033), OCT4 with age (*p* = 0.005) and tumor grade (*p* = 0.008) were noticed in the gastric cancer patient data. Expression of Nanog was higher in patients with new tumor development, whereas that of OCT4 was lower in the higher grade of gastric cancer. Higher expression of Nanog, but not the others, was significantly associated with a poor cumulative survival rate in Kaplan-Meyer survival analysis (*p* = 0.03) (Figure 1A–D). Associations of Nanog expression with new tumor development and poor survival of gastric cancer patients suggest a possibility that its expression might be implicated in the development of drug resistance and tumor stemness of gastric cancer. Meanwhile, expression of the pluripotency factors at the protein level was compared by western blotting in six gastric cancer cell lines (Figure 1E). KLF4 expression was noticeably high in SNU-601, compared to the other five cells that did not show a significant difference among them (*p* = 0.57 at ANOVA). Expression of Nanog was also highest in SNU-601, however, that of SOX2 was strongest in SNU-484. In contrast, OCT4 expression was not obvious in any of the six cell lines. Overall, pluripotency factor expression was divergent in the gastric cancer cell lines as shown in the prominent expression of KLF4 and Nanog in SNU-601 and of SOX2 in SNU-484.

### 3.2. Changes in the Pluripotency Factor Expression and Drug Sensitivity upon Spheroid Culture

**Figure 2:**
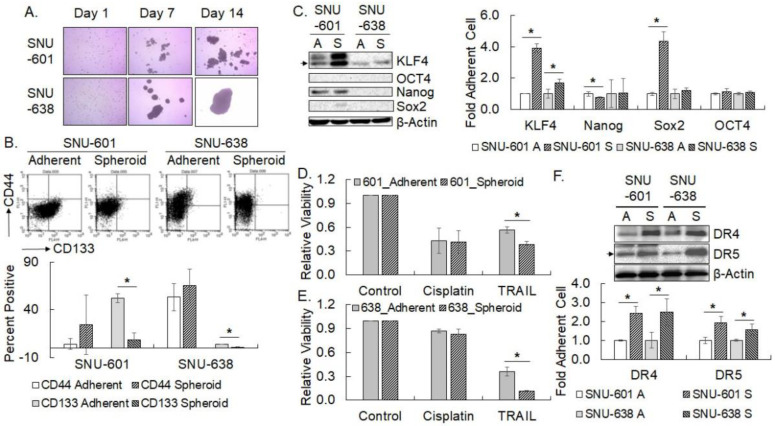
Expression of pluripotency factors and response to cisplatin or TRAIL treatment of spheroid-cultured gastric cancer cells. (**A**) Representative phase contrast images of nonadherent spheroid culture of SNU-601 and SNU-638 on ultralow attachment plates for 14 days. (**B**) Flow cytometric analysis of cell surface expression of CD44 and CD133. Corresponding quantification results in the lower panel are shown in the mean ± SD of two independent experiments. (**C**) Expression of indicated pluripotency factors in spheroid-cultured SNU-601 and SNU-638 for 14 days was examined by western blotting. Quantified results were normalized against β-Actin level and fold increase over adherent culture control is shown in the mean ± SD of three independent experiments. (**D**,**E**) Cell viability of the spheroid-cultured SNU-601 (**D**) and SNU-638 (**E**) treated with cisplatin (0.5 µg/mL) or TRAIL (50 ng/mL) for three days was measured by MTT assay. Data shown are the mean ± SD of relative viability normalized against untreated control of three independent experiments. (**F**) Western blot analysis of death receptor expression in the spheroid-cultured SNU-601 and SNU-638. Quantification results were normalized against β-Actin level and fold increase over adherent culture control is shown in mean ± SD of three independent experiments. ‘A’ stands for adherent culture and ‘S’ for spheroid culture. * represents *p* < 0.05.

Tumor spheroid culture has been applied to enrich tumor stem cells or stem cell-like cells with stem cell properties [7]. Preliminary studies showed a sharp contrast in the expression of KLF4 and Nanog, response to cisplatin and TRAIL, and spheroid-forming capacity between SNU-601 (KLF4/Nanog-high) and SNU-638 (KLF4/Nanog-low). Thence, in order to examine the pluripotency factor expression and drug response of spheroid-forming cells, SNU-601 and SNU-638 were cultured in anchorage-independent spheroid-forming conditions. While SNU-638 formed round-spheroid bodies of variable sizes (up to 1.9 mm in diameter) on days 7 and 14 of the spheroid culture, SNU-601 formed clumps of aggregated cells instead of typical spheroid bodies (Figure 2A). To verify the enrichment of gastric cancer stemoid cells by the tumor spheroid culture for 14 days, the expression of gastric cancer stem cell markers including CD44 and CD133 was examined by flow cytometry. CD133-high cells were significantly decreased by the spheroid culture of SNU-601 and SNU-638 cells, whereas CD44 expression showed a tendency to increase in both cell lines (Figure 2B). The expression of four pluripotency factors in the spheroid-cultured cells was examined by western blotting (Figure 2C). KLF4 expression was markedly increased in both SNU-601 and SNU-638 cells under the spheroid culture condition. Although less obvious in SNU-638 spheroid-forming cells, SOX2 expression was also enhanced in SNU-601 clump-forming cells. Meanwhile, the expression of Nanog was decreased in SNU-601 only, while that of OCT4 did not change noticeably by the spheroid culture. Next, the response of SNU-601 clump-forming cells and SNU-638 spheroid-forming cells to cisplatin or TRAIL treatment was measured by an MTT assay. TRAIL treatment significantly decreased the viability of both SNU-601 clump forming-cells and SNU-638 spheroid-forming cells compared with parental cells, whereas the response to cisplatin did not change in the spheroid-cultured SNU-601 and SNU-638 (Figure 2D,E). The expression of DR4 and DR5 TRAIL receptors was shown to be greatly elevated by the spheroid culture of both cells, which was consistent with the increased TRAIL sensitivity (Figure 2F). In summary, the spheroid culture of SNU-601 and SNU-638 enhanced the expression of KLF4 and SOX2 and decreased the surface expression of CD133. In addition, death receptor expression was elevated by spheroid culture, which might render the spheroid-cultured cells more susceptible to TRAIL over the parental cells grown in adherent culture conditions.

### 3.3. Pluripotency Factor Expression upon Cisplatin or TRAIL Treatment

**Figure 3:**
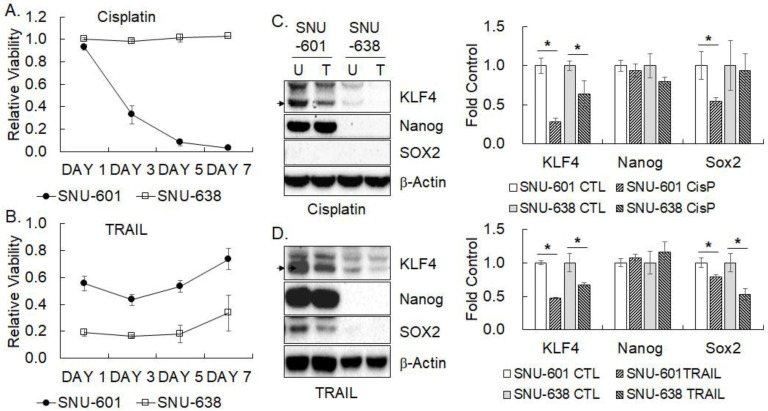
Expression of pluripotency factors in gastric cancer cells treated with cisplatin or TRAIL. (**A**, **B**) SNU-601 and SNU-638 cells grown in adherent culture condition were treated with cisplatin (0.5 µg/mL, (**A**)) or TRAIL (50 ng/mL, (**B**)) and cell viability was measured by MTT assay on indicated days after treatment. Data shown are the mean ± SD of relative viability normalized against untreated control of three independent experiments. (**C**,**D**) Protein levels of indicated pluripotency factors in the gastric cancer cells treated with cisplatin (0.5 µg/mL, (**C**)) or TRAIL (50 ng/mL, (**D**)) for three days were examined by western blotting. Quantified results were normalized against β-Actin level and fold increase over untreated control is shown in the mean ± SD of three independent experiments. U stands for untreated cells and T for treated ones. * represents *p* < 0.05.

SNU-601 and SNU-638 were treated with cisplatin (0.5 µg/mL) or TRAIL (50 ng/mL) for one, three, five, and seven days, and cell viability was measured by MTT assay. The viability of SNU-638 was maintained upon the cisplatin treatment compared to untreated control, while that of SNU-601 was decreased to 33% of untreated control on day three of cisplatin treatment and further decreased with time (Figure 3A). On the contrary, SNU-638 was very sensitive to TRAIL treatment showing less than 20% of untreated control on days one and three, whereas SNU-601 was moderately sensitive and maintained ~50% relative viability against the untreated control (Figure 3B). Interestingly, the viability of both SNU-601 and SNU-638 was decreased until day three, however, it began to recover after day five of TRAIL treatment. Changes in the expression of three pluripotency factors (KLF4, Nanog, and SOX2) except OCT4 were examined in the gastric cancer cells treated with cisplatin or TRAIL for 72 h by western blotting (Figure 3C,D). KLF4 expression was decreased by the treatment of cisplatin or TRAIL in the gastric cancer cells. In fact, decreased KLF4 expression was sustained until day seven (Supplemental Figure S1). The expression of SOX2 also significantly declined in SNU-601 upon cisplatin treatment and in both cells upon TRAIL treatment. In contrast, the expression of Nanog was not altered upon cisplatin and TRAIL treatments meaningfully.

### 3.4. Modulation of the Responses to Cisplatin and TRAIL by KLF4

**Figure 4:**
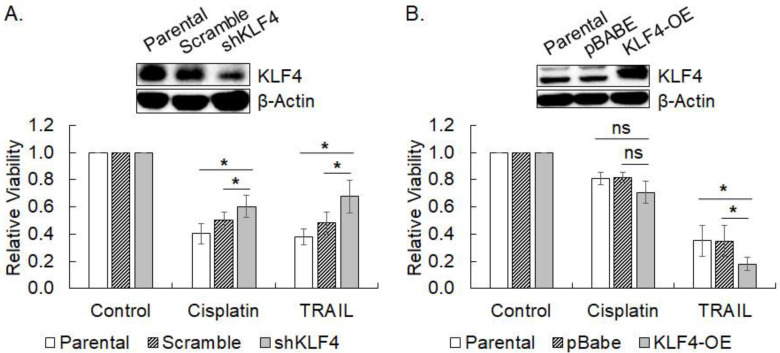
Changes in drug response by modulation of KLF4 expression. (**A**) KLF4 expression was silenced by shRNA targeting KLF4 (shKLF4) in SNU-601 (KLF4-high cells, *inset*). The viability of the cells treated with cisplatin (0.5 µg/mL) or TRAIL (50 ng/mL) for three days was measured by MTT assay. Data shown are relative viability normalized against untreated parental cells in mean ± SD of five independent experiments. (**B**) KLF4 was overexpressed by retroviral transduction of KLF4 cDNA (KLF-OE) in SNU-638 (KLF4-low cells, *inset*). Cell viability of the cells treated with cisplatin or TRAIL for three days was measured by MTT assay. Data shown are relative viability normalized against untreated parental cells in mean ± SD of five independent experiments. * represents *p* < 0.05.

Since the spheroid culture of SNU-601 and SNU-638 increased the KLF4 protein level and susceptibility to TRAIL, the effect of KLF4 expression on the response to cisplatin and TRAIL was investigated by measuring the cell viability of KLF4-silenced SNU-601 (KLF4-high) and KLF4-overexpressed SNU-638 (KLF4-low). Knockdown of KLF4 expression in SNU-601 cells by lentiviral transduction (pLKO.1-shKLF4) increased cell viability significantly against scrambled control by 29% upon cisplatin treatment (*p* = 0.018) and by 40% upon TRAIL treatment (*p* = 0.021) (Figure 4A). In contrast, overexpression of KLF4 in SNU-638 by retroviral infection (pBABE-puro-KLF4) decreased the cell viability of TRAIL-treated cells to 52% of vector only control (*p* = 0.016), while the viability of cisplatin-treated cells was not reduced so much as to declare it significant (Figure 4B). Overexpression of KLF4 also reduced the viability of KLF4-low SNU-484 and SNU-668 cells upon TRAIL treatment by 50% (*p* = 0.00002) and 55% (*p* = 0.00012) of vector only control, respectively (Supplemental Figure S2). Taken together, the KLF4 expression level appears to be associated with the TRAIL susceptibility of gastric cancer cells. Since the upregulation of KLF4 did not alter the response to cisplatin significantly, its association with cisplatin sensitivity could not be conclusive, yet.

### 3.5. Modulation of the Responses to Cisplatin and TRAIL by Nanog

**Figure 5:**
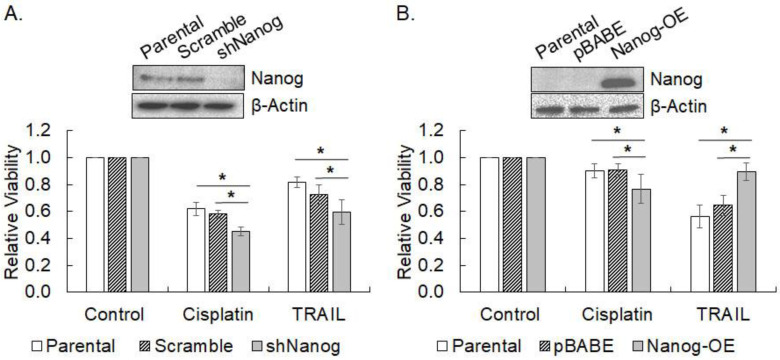
Changes in drug response by modulation of Nanog expression. (**A**) Nanog expression was silenced by shRNA targeting Nanog (shNanog) in SNU-601 cells (Nanog-positive cells, *inset*). Cell viability of the cells treated with cisplatin (0.5 µg/mL) or TRAIL (50 ng/mL) for three days was measured by MTT assay. Data shown are relative viability normalized against untreated parental cells in the mean ± SD of five independent experiments. (**B**) Nanog was overexpressed by retroviral transduction of Nanog cDNA (Nanog-OE) in SNU-638 cells (Nanog-negative cells, *inset*). Cell viability of the cells treated with cisplatin or TRAIL for three days was measured by MTT assay. Data shown are relative viability normalized against untreated parental cells in the mean ± SD of five independent experiments. * represents *p* < 0.05.

The effect of another pluripotency factor Nanog on the response to TRAIL and cisplatin treatments was also examined in Nanog-silenced SNU-601 (Nanog-high) and Nanog-overexpressed SNU-638 (Nanog-low) by MTT assay. Western blots showed the downregulation of Nanog in SNU-601 by transduction of shNanog lentivirus and the upregulation of Nanog in SNU-638 by the transduction of retrovirus with Nanog cDNA (Figure 5A and Figure 5B, respectively). Contrary to KLF4, the knockdown of Nanog significantly reduced the viability of cisplatin-treated SNU-601 cells by 22% (*p* = 0.0001, Figure 5A). However, a 13% viability reduction was also observed in Nanog-overexpressing SNU-638 cells treated with cisplatin (*p* = 0.031), compromising the specific effect of Nanog expression on cisplatin response (Figure 5B). In contrast, the downregulation of Nanog significantly diminished the viability of SNU-601 cells upon TRAIL treatment by 18% (*p* = 0.041) compared to the scrambled control (Figure 5A). In accord, the upregulation of Nanog significantly increased the viability of SNU-638 cells by 39% (*p* = 0.0004) over the vector only control (Figure 5B). These results strongly suggest that the Nanog expression level could influence the TRAIL response of the cancer cells.

### 3.6. KLF4 and Nanog on Spheroid Formation of SNU-601 and SNU-638

**Figure 6:**
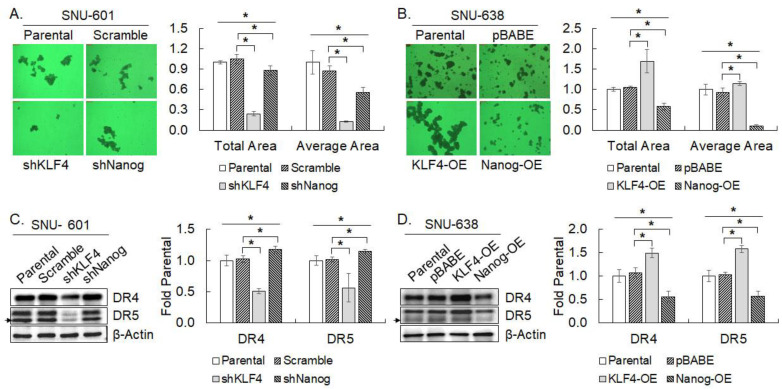
Changes in spheroid forming capacity and death receptor expression by modulation of KLF4 or Nanog expression. (**A**,**B**) Spheroid formation of KLF4- or Nanog-silenced SNU-601 (**A**) and KLF4- or Nanog-overexpressed SNU-638 (**B**). SNU-601 transduced with shKLF4 or shNanog lentivirus (**A**) and SNU-638 transduced with KLF4 or Nanog cDNA retrovirus (**B**) were cultured in nonadherent spheroid culture condition for 14 days. The total surface area taken by the spheroids and the area of the individual spheroid were measured with Image J. Data shown are representative phase contrast images of the spheroid culture, and fold increase in total area and average area over mock-transduced parental control in mean ± SD of three independent experiments. (**C**,**D**) Death receptor expression in KLF4- or Nanog-silenced SNU-601 (**C**) and in KLF4- or Nanog-overexpressed SNU-638 (**D**). Expression of DR4 and DR5 was examined by western blotting. The expression level of DR4 and DR5 was quantified and normalized against β-Actin level. Fold increase over mock-transduced parental control is shown in the mean ± SD of three independent experiments. * represents *p* < 0.05.

KLF4 expression was elevated in spheroid-cultured SNU-601 and SNU-638, while Nanog expression was decreased by the spheroid culture of SNU-601. Thus, the spheroid or clump-forming capacity was investigated in the cells in which the expression of KLF4 and Nanog was silenced and overexpressed, respectively, as above. KLF4 silencing in SNU-601 reduced both the total area and the average area taken by cell clumps extensively by 77% and 86%, respectively, compared to the scrambled control (Figure 6A). Accordingly, the overexpression of KLF4 in SNU-638 significantly increased the total area and the average area of the spheroids by 60% and 23%, respectively, compared to the vector only control (Figure 6B). Knockdown of Nanog in SNU-601 significantly decreased the total area and the average area taken by cell clumps, however, much less intensively than KLF4 silencing (16% and 37%, respectively, Figure 6A). Unexpectedly, however, Nanog overexpression in SNU-638 also decreased the total and average areas of the spheroids by 45% and 88%, respectively, which is much stronger than the effect of Nanog silencing (Figure 6B). These results indicate that the expression of KLF4 should influence the spheroid forming capacity of the gastric cancer cells, while the effect of Nanog expression on spheroid forming capacity appears nonspecific in essence.

### 3.7. KLF4 and Nanog on the Expression of DR4 and DR5

Since the expression of both KLF4 and Nanog was implicated in the response to TRAIL of SNU-601 and SNU-638, the expression of DR4 and DR5 was examined in the cells in which KLF4 and Nanog expression was modulated as above. Knockdown of KLF4 decreased expression of both DR4 and DR5 by 50% and 55%, compared to the scrambled control, respectively, whereas Nanog silencing increased that of DR4 and DR5 by 15% and 14%, respectively (Figure 6C). In accord, overexpression of KLF4 elevated DR4 and DR5 expression by 39% and 54% compared to the vector only control, respectively, while that of Nanog reduced the expression of DR4 and DR5 by 48% and 45%, respectively. These results suggest that altered TRAIL sensitivity upon the modulation of KLF4 or Nanog expression could result from corresponding changes in DR4 and DR5 expression.

## 4. Discussion

Selective stemness factors are known to be expressed in various cancer cells and are associated with the clinicopathological parameters and the prognosis of patients of diverse cancers, including gastric cancer [16]. The upregulation of Nanog was associated with new tumor emergence after initial treatment and worse prognosis of gastric cancer patients in this study. These results support a previous report that the overexpression of Nanog was positively correlated with an advanced clinical stage and a worse prognosis of gastric adenocarcinoma patients [34]. The association of Nanog expression with survival was also found in breast, colorectal, head and neck, lung, and ovarian cancers, which corroborates the clinical significance of Nanog expression in cancer progression [21]. However, a meta-analysis on the clinical significance of various stem cell markers failed to identify an association of Nanog expression with the clinical outcome of gastric cancer patients [35]. Taken together, the prognostic value of Nanog expression in gastric cancer remains to be firmly determined.

The expression of OCT4, SOX2, Nanog, and KLF4 was examined in six gastric cancer cell lines at the basal level and upon a spheroid culture. KLF4 expression was detected in all six cells at various level, however, the others expressed cell type dependently. Cell type-dependent expression of SOX2 was also reported previously [36]. The expression of KLF4 and SOX2 was increased upon the spheroid culture of SNU-601 and SNU-638, while Nanog expression was decreased slightly, significantly only in SNU-601 cells. Upregulation of KLF4, SOX2, and OCT4, as well as a mixed response in Nanog expression upon a spheroid culture, were also described in previous studies [17,36,37].

SOX2 knockdown inhibited the spheroid formation of gastric cancer cells [36,38]. Here, the effect of KLF4 and Nanog on the sphere-forming capacity of SNU-601 (KLF4/Nanog-high) and SNU-638 (KLF4/Nanog-low) cells was examined. Both the knockdown and overexpression of Nanog reduced the total and average areas taken by the spheroids, suggesting a nonspecific effect of Nanog on spheroid formation. Taking these results together with the modest change in Nanog expression only in SNU-601, the role of Nanog in the spheroid formation of these cells appears to be limited. In contrast, KLF4 silencing decreased spheroid formation, whereas the upregulation of KLF4 increased it. Considering the upregulation of KLF4 in spheroid culture together with these results, KLF4 seems to play a role in spheroid formation. KLF4 stimulates stemness and mesenchymal properties of colorectal cancer stem cells by the activation of the TGF-β1 pathway, which might be involved in the KLF4-associated spheroid formation of the gastric cancer cells [39].

The expression of pluripotency factors in response to cisplatin treatment, and changes in drug response by spheroid culture or by modulation of KLF4 and Nanog expression were analyzed in SNU-601 (cisplatin-sensitive) and SNU-638 (cisplatin-resistant). Both KLF4 and SOX2 levels were decreased by cisplatin treatment, while Nanog expression was not changed significantly. Response to cisplatin treatment was not altered in spheroid-cultured cells or by the modulation of Nanog expression meaningfully. Both knockdown and upregulation of Nanog expression nonspecifically lowered cell viability of cisplatin-treated cells. In contrast, the knockdown of KLF4 raised the cell viability of cisplatin-treated cells. Although a statistical significance was compromised, a reduction in viability was observed in KLF4-overexpressing SNU-638 cells, which corresponded to the changes shown in KLF4-silenced cells. Reduction in KLF4 expression increased cell viability in SNU-1, while its upregulation in SNU-601 decreased cisplatin sensitivity [40]. Moreover, KLF4 upregulates the Bcl-2-interacting killer (BIK), which promotes cisplatin-induced apoptosis [41]. Taken together, both results suggest a role of KLF4 expression in the cisplatin response of gastric cancer cells. 

Pluripotency factor expression in response to TRAIL treatment and changes in drug response by spheroid culture or by modulation of KLF4 and Nanog expression were also analyzed in SNU-601 (moderate sensitivity to TRAIL) and SNU-638 (TRAIL-sensitive). As in the cisplatin-treated cells, both KLF4 and SOX2 levels were decreased at TRAIL treatment, while Nanog expression was not altered significantly. Interestingly, TRAIL sensitivity was increased in spheroid-cultured cells and KLF4-overexpressing and Nanog-silenced cells. KLF4 expression seemed to be positively associated with TRAIL susceptibility, whereas Nanog expression affected it in the opposite way. The expression of DR4 and DR5 was increased or decreased with corresponding changes in TRAIL responsiveness. 

Cancer stem cells have been known to manifest TRAIL resistance by the upregulation of antiapoptotic molecules and the activation of survival signals, although TRAIL resistance can be overcome by combined treatment with various small molecules [32]. Spheroid-cultured gastric cancer cells showed a higher TRAIL sensitivity than those cultured in the adherent conditions in this study. Considering that the spheroid culture of cancer cells is known to enrich stem cell-like cells, increased TRAIL sensitivity of spheroid-cultured gastric cancer cells is an unexpected result [7,32]. However, HTC-15 cells from soft agar-cultured colonies (CD44-high) were found more sensitive to TRAIL than the parental cells (CD44-low) [42]. In addition, CD44-positive HTC-15 cells were significantly more sensitive to TRAIL than CD44-negative cells. The increased TRAIL sensitivity in soft agar colony cells and CD44-positive cells was accompanied by the upregulation of DR4 and DR5. Accordingly, the enhanced TRAIL susceptibility of the spheroid-cultured gastric cancer cells seems to be associated with a tendency of CD44 upregulation and an increased expression of DR4 and DR5 upon the spheroid culture of the gastric cancer cells. On the other hand, TRAIL susceptibility of breast cancer stem cells was dependent on the cytoplasmic cFLIP level that was positively correlated with the CD133 level [43,44]. The spheroid culture of the gastric cancer cells decreased the CD133 level significantly. The reduction in CD133 might be associated with a concomitant decrease in cFLIP level, which might result in increased TRAIL susceptibility of the spheroid-cultured gastric cancer cells.

The overexpression of KLF4 and knockdown of Nanog upregulated DR4/DR5 expression and reduced the viability of the gastric cancer cells treated with TRAIL. KLF4 has been known to promote apoptosis of cancer cells in various ways, including upregulation of NOXA and suppression of the inhibitor of the apoptosis-stimulating protein of p53 (iASPP) expression [13]. In contrast, Nanog suppressed apoptosis through the regulation of p53 and its downstream genes such as Bax and Gadd45a [45]. However, the direct regulation of TRAIL-induced apoptosis and DR4/DR5 expression by KRF4 and Nanog has not been studied extensively. TRAIL-induced apoptosis was enhanced by various molecules that upregulated DR4 and/or DR5 expression in a p53-dependent way [46,47]. Silencing of Nanog expression promoted apoptosis of T-cell acute lymphoblastic leukemia in a p53-dependent way [48]. Thus, it is possible that p53, upregulated in Nanog-silenced cells, might cause an increased expression of the death receptors. Although KLF4 is a target of p53, however, it inhibits the p53-dependent apoptotic pathway by the suppression of Bax expression and promotes growth inhibition by recruiting p53 to the CDKN1A promoter [49]. In addition, direct regulation of DR4 and DR5 expression by KLF4 has not been described yet [50], which suggests a novel and probably indirect pathway for the upregulation of DR4 and DR5 in KLF4-overexpressing cells.

KLF4 expression was lower in the late and advanced stage of gastric cancer and its expression was inversely correlated with patients’ prognosis [19]. Stem cell-enriching spheroid culture of the gastric cancer cells increased KLF4 expression and enhanced TRAIL sensitivity with concomitant upregulation of DR4 and DR5. KLF4 overexpression upregulated DR4 and DR5 expression and increased TRAIL sensitivity. Taken together, KLF4 is supposed to play a critical role in survival, especially against TRAIL treatment of gastric cancer cells and in gastric cancer progression. Meanwhile, SNU-601 was less sensitive to TRAIL treatment, even though the KLF4 expression of SNU-601 was found to be much higher than that of SNU-638. This result would not be compatible with the suggested role of KLF4 in the regulation of TRAIL sensitivity. However, SNU-601 expressed Nanog higher than the other cells, including SNU-638. Since Nanog regulated DR4/DR5 expression and TRAIL sensitivity in a contrary way to KLF4, it is feasible that Nanog expression in SNU-601 could compensate for the effect of KLF4 on TRAIL sensitivity. Therefore, it is necessary to consider the opposite effect of KLF4 and Nanog on the TRAIL response of gastric cancer cells.

## 5. Conclusions

In summary, an association of pluripotency factor expression with clinicopathological parameters of gastric cancer patients and the effect of Nanog and KLF4 expression on the spheroid forming capacity and response to cisplatin and TRAIL treatments of gastric cancer cells are described in this study. Nanog expression was associated with the emergence of new tumors after initial treatment and the worse prognosis of gastric cancer patients. KLF4 and Nanog were expressed high in SNU-601, whereas SOX2 was high in SNU-484. The expression of KLF4 and SOX2 was increased by the spheroid culture of SNU-601 and SNU-638. In addition, the spheroid culture of them enhanced TRAIL-induced viability reduction, which was accompanied by the upregulation of DR4 and DR5. Modulation of Nanog expression did not affect spheroid-forming capacities, but its expression was inversely correlated with DR4 and DR5 expression and TRAIL sensitivity. By contrast, KLF4 overexpression increased spheroid formation, susceptibility to cisplatin and TRAIL treatments, and DR4/DR5 expression, and vice versa. Both Nanog and KLF4 altered death receptor expression and TRAIL sensitivity in the gastric cancer cells, however, in the opposite way. Direct regulation of death receptor expression and TRAIL response by KLF4 and Nanog has not been well documented and the regulatory mechanism of the process remains to be elucidated.

## Figures and Tables

**Table 1:** Association of clinicopathological parameters and expression of four pluripotency factors in gastric cancer patients. Probabilities were calculated by the Wilcoxon rank sum test.

Pathol. Parameter	Category	KLF4	NANOG	OCT4	SOX2
Sex	Male (267)-Female (147)	0.914	0.089	0.314	0.065
Age	Dn 65 (184)-Up 65 (225)	0.842	0.129	*0.005*	*0.033*
Race	White (259)-Asian (87)	0.072	0.088	0.375	0.757
Hist Dx	Intestinal (176)-Diffused (69)	0.971	0.806	0.183	0.784
Grade	G1/2 (159)-G3 (246)	0.701	0.930	*0.008*	0.299
T Status	T1/2 (110)-T3/4 (295)	0.062	0.213	0.880	0.317
N Status	N0 (122)-N1~3 (273)	0.378	0.580	0.780	0.094
M Status	M0 (367)-M1 (27)	0.251	0.399	0.420	0.321
Tumor Stage	Stage I/II (179)-III/IV (210)	0.700	0.767	0.368	0.320
Treat Outcome	Rem-P. Rem (137)/S-Prog (49) *	0.689	0.588	0.285	0.391
New Tumor	Yes (45)/No (171)	0.719	*0.027*	0.203	0.090

* ‘Rem-P. Rem’ for remission-partial remission; S-Prog for stable-progressed. Numbers in the parenthesis are numbers of patients. The italicized/bold are *p* < 0.05.

## Data Availability

The data will be available on request.

## Supplementary Materials

The following supporting information can be downloaded at: https://www.mdpi.com/article/10.3390/cimb45010018/s1, Supplemental Figure S1. Expression of KLF4 in SNU-601 and SNU-638 treated with cisplatin or TRAIL for seven days: Supplemental Figure S2. Modulation of TRAIL response by overexpression of KLF4 in SNU-484, SNU-638, and SNU-668.
