# Supplementary material for: Modulation of Spheroid Forming Capacity and TRAIL Sensitivity by KLF4 and Nanog in Gastric Cancer Cells
Source: Curr Issues Mol Biol. 2022 Dec 30;45(1):233–48. doi: 10.3390/cimb45010018 (PMC9857986; doi:10.3390/cimb45010018)
Supplement: Supplementary file 1 [file cimb-45-00018-s001.zip › cimb-2040320-supplementary.pdf]

Supplemental Figure S1. Expression of KLF4 in SNU-601 and SNU-638 treated with cisplatin or TRAIL for seven days.

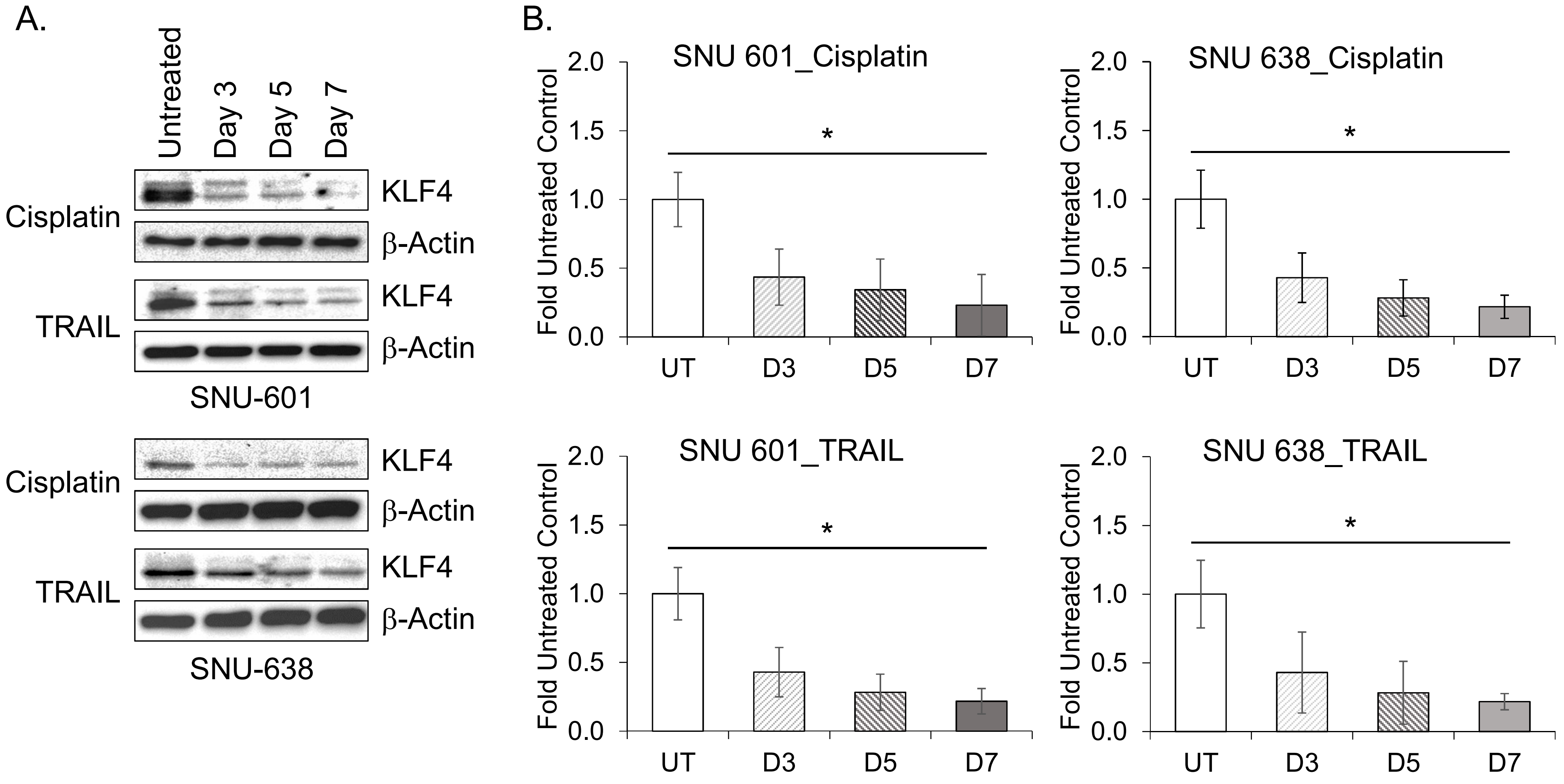

A. KLF4 expression in SNU-601 and SNU-638 treated with cisplatin (0.5 μg/ml) or TRAIL (50 ng/ml) for indicated period were examined by western blotting. **B**. Quantified results were normalized against β-Actin level and fold increase over untreated control is shown in mean ± SD of three independent experiments. * represents $p<0.05$.

Supplemental Figure S2. Modulation of TRAIL response by overexpression of KLF4 in SNU-484, SNU-638 and SNU-668

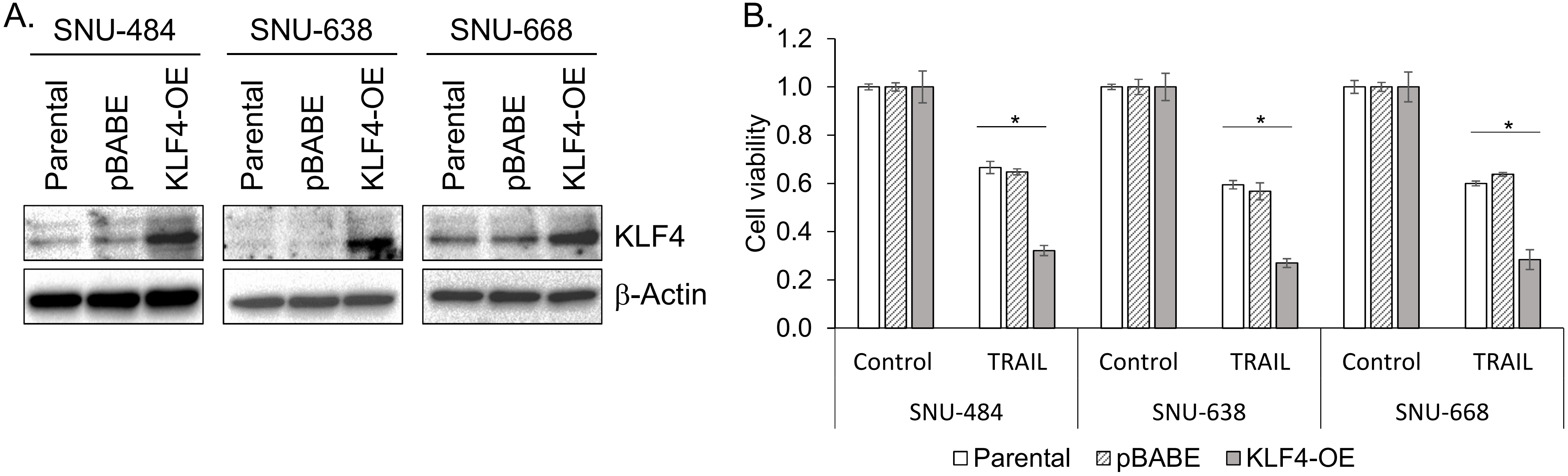

A. Overexpression of KLF4 by retroviral transduction of KLF4 cDNA (KLF-OE) in SNU-484, SNU-638 and SNU-668 was verified by western blotting. **B**. Cell viability of the KLF4-overexpressing cells treated with cisplatin (0.5 μg/ml) or TRAIL (50 ng/ml) for three days was measured by MTT assay. Data shown are relative viability normalized against untreated parental cells in mean ± SD of three independent experiments. * represents $p<0.05$.
